# High Moesin Expression Is a Predictor of Poor Prognosis of Breast Cancer: Evidence From a Systematic Review With Meta-Analysis

**DOI:** 10.3389/fonc.2021.650488

**Published:** 2021-11-26

**Authors:** Xiaoli Hu, Yang Liu, Zhitong Bing, Qian Ye, Chengcheng Li

**Affiliations:** ^1^ Department of Medical Physics, Institute of Modern Physics, Chinese Academy of Sciences, Lanzhou, China; ^2^ College of Life Sciences, University of Chinese Academy of Sciences, Beijing, China; ^3^ Key Laboratory of Heavy Ion Radiation Medicine of Chinese Academy of Sciences, Lanzhou, China; ^4^ Key Laboratory of Heavy Ion Radiation Medicine of Gansu Province, Lanzhou, China; ^5^ Advanced Energy Science and Technology Guangdong Laboratory, Huizhou, China; ^6^ School of Stomatology, Lanzhou University, Lanzhou, China; ^7^ The First School of Clinical Medicine, Lanzhou University, Lanzhou, China

**Keywords:** MSN, lymph node metastasis, prognosis, breast cancer, meta-analysis

## Abstract

Owing to metastases and drug resistance, the prognosis of breast cancer is still dismal. Therefore, it is necessary to find new prognostic markers to improve the efficacy of breast cancer treatment. Literature shows a controversy between moesin (MSN) expression and prognosis in breast cancer. Here, we aimed to conduct a systematic review and meta-analysis to evaluate the prognostic relationship between MSN and breast cancer. Literature retrieval was conducted in the following databases: PubMed, Web of Science, Embase, and Cochrane. Two reviewers independently performed the screening of studies and data extraction. The Gene Expression Omnibus (GEO) database including both breast cancer gene expression and follow-up datasets was selected to verify literature results. The R software was employed for the meta-analysis. A total of 9 articles with 3,039 patients and 16 datasets with 2,916 patients were ultimately included. Results indicated that there was a significant relationship between MSN and lymph node metastases (*P* < 0.05), and high MSN expression was associated with poor outcome of breast cancer patients (HR = 1.99; 95% CI 1.73–2.24). In summary, there is available evidence to support that high MSN expression has valuable importance for the poor prognosis in breast cancer patients.

**Systematic Review Registration:**

https://inplasy.com/inplasy-2020-8-0039/.

## 1 Introduction

Breast cancer is one of the most common malignant cancers among women and it is a huge threat for them (1, 2). In 2020, it was estimated that there would be 281,550 new breast cancer cases and 43,600 deaths in the USA (https://seer.cancer.gov/statfacts/html/breast.html). Even though there are many therapies for breast cancer, most treatment plans include a combination of surgery, radiation, hormone therapy, chemotherapy, and targeted therapies (protein inhibitors, antibodies, and immunotherapy); however, the prognosis of breast cancer is poor (3, 4). Therefore, discovering a valuable prognostic biomarker to guide clinical therapy to improve the prognosis and quality of life of the patient is desperately needed.

Moesin (MSN), one of the ezrin–radixin–moesin (ERM) family of proteins, was isolated from bovine uterus. MSN is abundant in smooth muscle cells and exists in actin-rich cell surface structures such as microvilli, microspikes, membrane ruffles, and adhesion junctions (5, 6). MSN has three important domains: an ~300 residue N-terminal FERM domain, an ~200 residue α-helix linker domain, and an ~100 residue positively charged C-terminal tail domain that contains an F-actin binding site and a conserved threonine residue (7). MSN can switch between closed (inactive) and open (active) conformation. This homeostasis is modulated *via* a reversible intramolecular interaction between the N-terminal (FERM/NERMAD) domain and the C-terminal (C-ERMAD) domain in order to form a folded conformation that masks their functional sites (8, 9). When MSN acts as a cross-linker, the FERM domain separates itself from the tail, and the C-terminal domain can be phosphorylated by Rho-kinase or protein kinase C, allowing its interaction with F-actin (10, 11). Some studies showed that the activation state of MSN contributed to cell metastasis (12–14).

The mechanisms of tumor metastasis are complex. After undergoing a series of steps, tumor cells colonize and adapt to distal tissues (15). Epithelial–mesenchymal transition (EMT) is a key process for tumor cells to gain invasive capabilities. Tumor cells lose their polarity and change the way they interact with each other. Most importantly, these changes are accompanied by actin cytoskeleton rearrangements and lead to the formation of membrane protrusions (16–18). During EMT, changes of cell adhesion molecules have an effect on tumor metastasis; for example, the expression of N-cadherin is increased and the expression of E-cadherin is reduced. A previous study has demonstrated that the interruption of E-cadherin expression could lead to early invasion and metastasis (18, 19). Invadopodia are membrane protrusions formed by tumor cells, which could modify the extracellular matrix (ECM) cross-linked networks and promote tumor metastasis (20). Activated MSN participates in these metastatic steps. A study showed that elevated MSN expression reduces the level of E-cadherin/p120-catenin adhesion interaction complex, which could break up cell–cell adhesion (21). Moreover, activated MSN can interact with extracellular matrix protein 1 (ECM1) facilitating the formation of invadopodia (22). In addition, a study reported that activated MSN recruits sodium/hydrogen/exchanger 1 (NHE1) protein, leading to actin polymerization through the interaction between cortactin and cofilin (23). In this step, membrane type 1-matrix metalloproteinase (MT1-MMP) is recruited to degrade the ECM (24, 25). Lymph node metastasis is considered a hallmark of tumor progression (26). Kobayashi et al. (27) elucidated that lymph node metastasis was related with expression patterns of MSN in oral squamous cell carcinoma (OSCC), and most metastatic tumors showed a cytoplasmic distribution pattern. All the above studies suggest that MSN expression is closely related to tumor invasion and metastasis.

There is accumulating evidence suggesting that MSN expression could be an unfavorable prognostic molecular biomarker in several types of tumors. Barros et al. (10) showed that strong MSN expression had a negative effect on overall survival (OS) (*P* = 0.024) of OSCC patients in stages II and III. Also, they showed that MSN expression could enhance the risk of death (*P* = 0.022). Liang et al. (28) also reported that MSN expression was closely related with poor prognosis in pancreatic cancer. A recent study showed that MSN expression was correlated with a more aggressive phenotype and worse prognosis of OSCC (21). Moreover, it has been reported that MSN plays a significant role in cell metastasis in glioblastoma and hepatocellular carcinoma (13, 29). High MSN expression promoted migration not only in different types of tumors but also in breast cancer cells (30, 31). Furthermore, MSN interacted with other molecules promoting tumor invasion and metastasis (9). However, the survival outcome of breast cancer patients with MSN expression remains inconsistent (32, 33). This paper aims to systematically review the association of MSN expression with breast cancer and, using quantitative synthesis, to assess if high (positive) MSN expression was related with worse outcome of patients with breast cancer.

## 2 Materials and Methods

### 2.1 Protocol Registration and Search Strategy

This present study followed the Preferred Reporting Items for Systematic Reviews and Meta-Analysis (PRISMA) guidelines (34). The protocol of this present study is available at INPLASY.COM (registration number *INPLASY202080039, DOI number 10.37766/inplasy2020.8.0039).* We conducted an integrated search in Web of Science, Embase, Cochrane Library, and PubMed. In the present study, we searched the literature based on the following terms: (“moesin” OR “membrane-organizing extension spike protein” OR “Msn protein” OR “moesin protein” OR “MSN protein”) AND (“breast cancer*” OR “Breast Neoplasm*” OR “Breast Tumor*” OR “Breast Cancer*” OR “Mammary Cancer*” OR “Malignant Neoplasm of Breast” OR “Breast Malignant Neoplasm*” OR “Malignant Tumor of Breast” OR “Breast Malignant Tumor*” OR “Cancer of Breast” OR “Cancer of the Breast” OR “Mammary Carcinoma*” OR “Human Mammary Carcinoma*” OR “Human Mammary Neoplasm*” OR “Breast Carcinoma*”) (the detailed search strategy is shown in **Table S1**). Moreover, in order to ensure the integrity of the data, we carried out a reduplicative search on June 23, 2020.

### 2.2 Eligibility Criteria and Study Selection

#### 2.2.1 Inclusion Criteria and Exclusion Criteria

The included literature met the following criteria: a) publications investigated the association of MSN expression with clinical prognosis of breast cancer patients; b) patients were divided into high (positive) and low (negative) MSN expression groups in original articles; c) research studies were published in English or Chinese; and d) survival outcomes provided in the original articles included OS, progression-free survival (PFS), relapse-free survival/recurrence-free survival (RFS), cancer-specific survival (SS), metastasis-free survival (MFS), or disease-free survival (DFS).

All studies for exclusion met these criteria: a) publications described other ERM family of proteins (ezrin or radixin), b) studies investigated the correlation between MSN and biological mechanisms but not exploring the relationship between MSN and the clinical prognosis, and c) duplicate publications.

#### 2.2.2 Study Selection

All of the records were imported in EndNote X9 and two researchers independently selected the literature by screening titles and abstracts. Further screening was done by reading the full text. Disagreements were resolved after discussion with all of the authors.

### 2.3 Assessment of Reporting Quality

Three independent researchers conducted a quality assessment according to the Reporting Recommendations for Tumor Marker Prognostic Studies (REMARK) guidelines (35). Based on the REMARK guidelines and a previous study (36), we adapted six checklist items in our present study: a) patient samples, b) clinical data of the cohort, c) assay methods, d) prognostics, e) statistical analysis, and f) classical prognostic factors (**Table S2**). Disagreements were resolved after a consensus-based discussion with all of the authors.

### 2.4 Data Extraction

Two researchers independently extracted significant data, and ultimate results were obtained after reaching a consensus with a senior researcher. The main information is as follows: name of researchers, country of origin, publication date, age, the number of patients, detection methods of MSN and follow-up time, breast cancer types, tumor size, histological grade, TNM stage, RFS, OS, SS, MFS, DFS, and PFS. Because the values of hazard ratio (HR) and the corresponding 95% confidence interval (CI) were not reported in the included articles, to explore the relationship between high (positive) MSN and breast cancer, we used Tierney’s method (37) to calculate the HRs for the included studies.

### 2.5 Statistical Analysis

Based on Tierney’s method (37), the HRs and 95% CIs were calculated and further heterogeneity test was conducted. If *P <*0.05 and/or *I* ^2^ >50%, there was significant heterogeneity, and the random-effect model was used to calculate the pooled HR; on the contrary, if there was no significant heterogeneity, the fixed-effect model was used. The value of HR >1 and of the diamond does not overlap with the invalid line, suggesting that high MSN expression was statistically significant for poor prognosis in breast cancer patients.

### 2.6 Meta-Analysis of the Validation Datasets

To further verify the literature results, the GEO database was applied for validation. In this study, we used the KM plotter web tool to collect gene expression and clinical information data of breast cancer (38). A total of 16 datasets were obtained after screening the datasets with more than 90 samples. The prognosis of MSN was analyzed in 2,916 breast cancer patients from the GEO datasets.

### 2.7 Kruskal–Wallis Test

The Kruskal–Wallis test was used to investigate the relationship between clinicopathological parameters and MSN expression. *P <*0.05 was considered statistically significant. The clinicopathological data were downloaded from the TCGA-BRCA database (https://portal.gdc.cancer.gov/). The clinicopathological parameters (*n* = 622) included age at diagnosis, estrogen receptor (ER) status, progesterone receptor (PR) status, HER2 status, histological type, count of lymph node examined, and AJCC stage.

## 3 Results

### 3.1 Literature Selection and Characteristics of Studies

**Figure 1:**
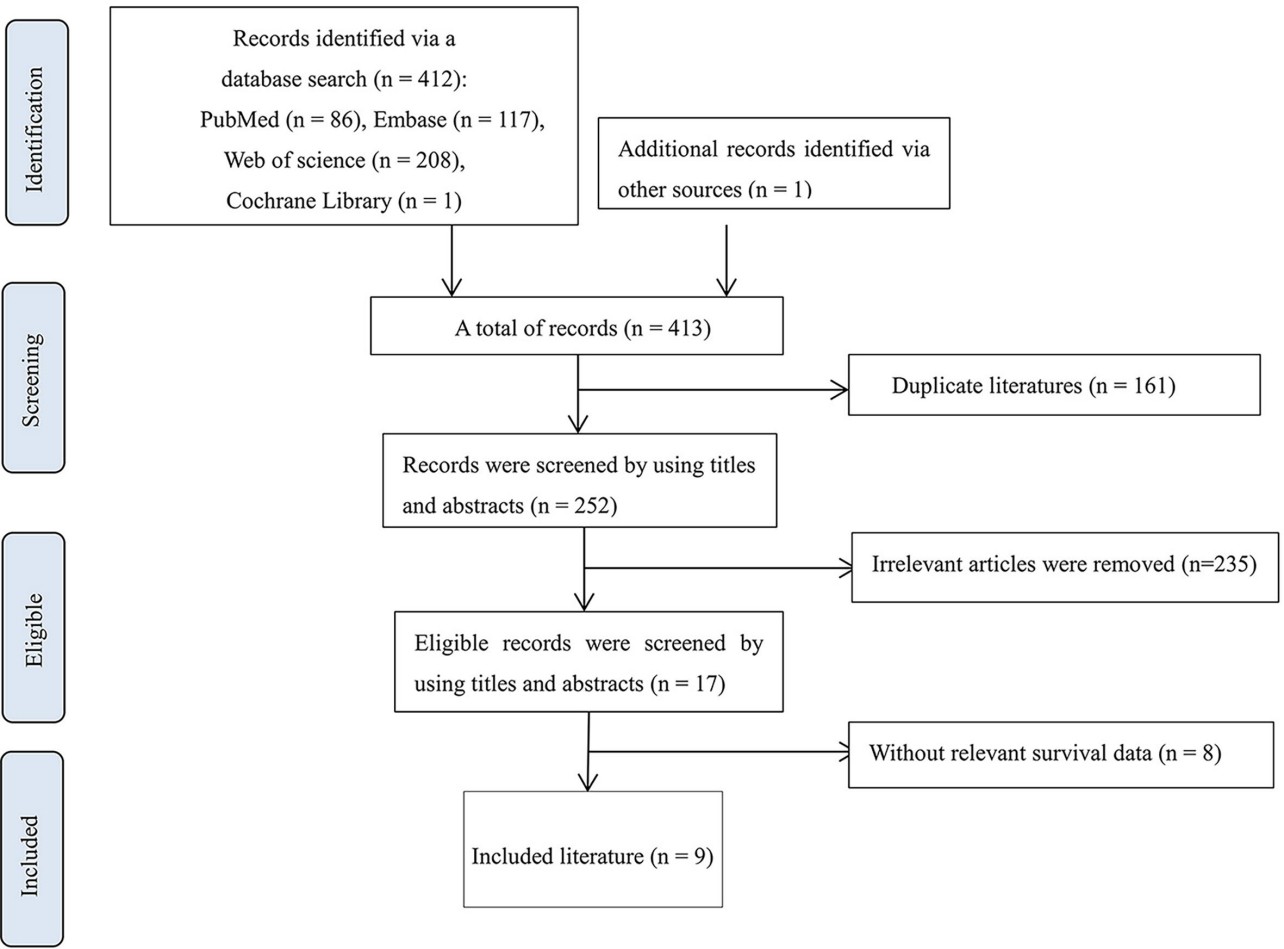
Flowchart representing the systematic literature search on MSN and breast cancer.

In total, the database search yielded 413 citations. Then, 161 duplicate literatures were removed, and 235 irrelevant records were excluded by screening titles and abstracts. Eight articles showed the correlation between MSN and biological mechanisms but did not describe the relationship between MSN expression and clinical prognosis (**Table S3**). Finally, nine eligible records were included (33, 39–46) (**Table S4**). The literature selection process is shown in **Figure 1**.

The publication years of the included studies were from 2004 to 2020. The characteristics of the citations are shown in **Table 1**, and the patient cohorts were from France (*n* = 3), Poland (*n* = 1), Australia (*n* = 1), and China (*n* = 4). Three out of the nine articles described the average age of the patients, which ranged from 20 to 94 years old. The studies enrolled 3,039 cases (ranging from 104 to 1200 per study). As for the detection methods of MSN, eight records used immunohistochemistry (IHC). Besides, four studies described tumor size, and two out of nine described the TNM stage. The cutoff value of MSN expression is shown in **Table 1**.

**Table 1:** Characteristics of the included articles.

Authors	Country	Year	No. of patients	Age, mean (range)	Type	Sample source	Assay	Tumor size (cm)	TNM stage	Cutoff value
Chotteau-Lelièvre (33)	France	2004	364	58 (26–90)	BCs	–	RT-PCR	≤2 (*n* = 29), 2 to 5 (*n* = 234), ≥5 (*n* = 88)	–	0.05
Charafe-Jauffret (39)	France	2007	482	59 (25–94)	IBCs, MBCs, and SBCs	TMA	IHC	≤2 (*n* = 204), >2 (*n* = 276)	–	0
Charpin (40)	France	2009	1200	–	BCs	FFPE tissues	IHC	–	–	16.4
Donizy (41)	Poland	2011	–	–	BCs	FFPE tissues	IHC	–	–	3
Wang (42)	China	2012	144	–	IDCs, ILCs, MCs, MCCs, IPCs, and MCBs	FFPE tissues	IHC	–	–	≥0.1
Li (43)	Australia	2014	–	–	BCs	FFPE tissues	IHC	–		–
Pei (44)	China	2016	104	–	BIC-NST, BDCIS, and NAT	FFPE tissues	IHC	≤2 (*n* = 27), 2 to 5 (*n* = 46), ≥5 (*n* = 31)	I + II (*n* = 63), III + IV (*n* = 41)	5
Yu (45)	China	2019	450	51 (20–82)	BCs and BF	FFPE tissues	IHC	≤2 (*n* = 159), 2 to 5 (*n* = 188), ≥5 (*n* = 57)	I (*n* = 106), II (*n* = 247), and III (*n* = 51)	15
Qin (46)	China	2020	295	–	TNBC and non-TNBC	–	IHC	–	–	–

BCs, breast cancers; IBCs, invasive BCs; MBCs, medullary BCs; BRCA1-BCs, BRCA1-associated breast cancers; SBCs, sporadic breast cancers matched on the age of patients; IDCs, invasive ductal carcinomas; ILCs, invasive lobular carcinomas; IPCs, invasive papillary carcinomas; MCBs, metaplastic carcinoma of the breast; MCCs, mucinous carcinomas; BIC-NST, breast invasive carcinoma of no specific type; BDCIS, breast ductal carcinoma in situ; NAT, normal adjacent tissues; BF, breast fibroadenoma; TMA, tissue microarray; FFPE, formalin-fixed, paraffin-embedded; OS, overall survival; RFS, relapse-free survival/recurrence-free survival; DFS, disease-free survival; MFS, metastasis-free survival; NS, not significant; RR, relative risk; ND, no data; IHC, immunohistochemistry; –, not reported.

### 3.2 Quality Assessment

Two records fulfilled all the REMARK criteria (33, 45). Three studies lacked one item (39, 42, 44). The study of Donizy et al. (41) lacked two items. One literature met three items (40), and two records only met two items (43, 46), which are shown in **Table 2**.

**Table 2:** Evaluation criteria used to assess the quality of the records.

Authors	Item 1	Item 2	Item 3	Item 4	Item 5	Item 6	Number of conforming items
Chotteau-Lelièvre (33)	√	√	√	√	√	√	6
Charafe-Jauffret (39)	√	√	√	√		√	5
Charpin (40)	√		√			√	3
Donizy (41)	√	√		√		√	4
Wang (42)	√	√	√	√		√	5
Li (43)				√		√	2
Pei (44)	√	√	√	√		√	5
Yu (45)	√	√	√	√	√	√	6
Qin (46)				√		√	2

The criteria were adapted from the Reporting Recommendations for Tumor Marker Prognostic Studies (REMARK) guidelines (35).

### 3.3 The Cutoff Values

The included studies applied different ways to detect the MSN expression, namely, a) a real-time one-step reverse transcription-PCR assay to quantify MSN expression and b) IHC. Immunoreactive scoring (IRS) and the method of stain area × stain intensity were used to determine the cutoff value of MSN expression. Chotteau-Lelièvre et al. (33) took 0.05 as the threshold value; the score <0.05 was regarded as “low expression,” and the opposite was high expression (besides, the article of Chotteau-Lelièvre et al. reported that 0.04 also could classify the expression of MSN). Charafe-Jauffret et al. (39) regarded that the value of quick score (QS) (47) superior to 0 was positive. Charpin et al. (40) defined 16.4 as the optimal threshold of MSN expression. Donizy et al. (41) used the IRS developed by Remmele to define the expression of MSN, and IRS ≥3 was an overexpression of MSN. Wang et al. (42) regarded that cases with cytoplasmic and/or membranous staining against MSN in 10% or more of tumor cells were positive. The cutoff value of MSN expression in the research of Li et al. (43) was unclear. Pei et al. (44) used the total points (stain area × stain intensity) ≥5 to represent the high expression and the total points ≤4 to represent the low expression. Yu et al. (45) selected 15.0 (IHC score) as the cutoff score, where IHC score >15.0 was the “high expression,” and IHC score ≤15.0 was the low expression. In the study of Qin et al. (46), there was also no description of MSN cutoff. These values are shown in **Table 1**.

### 3.4 MSN Expression and Clinicopathological Parameters

According to Pei et al. (44), the age of patients has no significant correlation with MSN expression (*P* > 0.05). However, Yu et al. (45) found that high MSN expression was related with the age at diagnosis of patients. For tumor size, it had no significant correlation with MSN expression (44, 45). As for the histological grade, one article clearly indicated that high histological grade was strongly correlated with MSN expression (*P* < 0.05) (42). Charafe-Jauffret et al. (39) showed that SBR grade was significantly correlated with MSN expression (*P* = 1.14E-08). One study showed that histological grade has no significant correlation with MSN expression (*P* > 0.05) (45). Another article showed that there was no significant correlation between grade I and grade II (*P* > 0.05), but grade III MSN expression was higher than grade I (*P* < 0.05) (44). Another six records did not show the correlation between MSN and histological grade (33, 39–41, 43, 46). Tumor cells often invade lymph nodes. The high expression of MSN IRS was strongly associated with lymph node metastases (*P* = 1.00e-05) (41). MSN expression had a significant correlation with positive node metastasis (*P* < 0.0001) (45).

One research from France showed that MSN expression was negatively correlated with ER (*P* = 0.019, *r* = −0.124), human epidermal growth factor receptor 3 (HER3) (c-erbB-3; *P* = 0.01, *r* = −0.135), and HER4 (c-erbB-4; *P* = 0.003, *r* = −0.154), but it was positively correlated with epidermal growth factor receptor (EGFR) (*P* < 0.001, *r* = 0.296) (33). In addition, Yu et al. (45) showed that MSN expression was significantly higher in ER-negative or PR-negative tumors than in ER-positive or PR-positive tumors (*P*_ER_ = 0.008, *P*_PR_ = 0.026). Wang et al. (42) showed that compared with non-triple negative breast cancer, there was a significantly higher MSN expression of patients with the triple−negative phenotype (*P* < 0.001). Since the original articles did not show the HRs and 95% CI of MSN and clinicopathological parameters, we did not merge relevant data.

### 3.5 MSN Expression and Patient Outcomes

In **Table 3**, there were five articles that described OS (33, 42, 44–46), three records that elucidated RFS (33, 43, 45), two articles that exhibited SS (39, 41), two that showed MFS (39, 43), and one that showed DFS (41). When multivariate analyses included some parameters such as prognostic grade, tumor size, and ER/PR status, MSN expression could be considered as a prognostic biomarker (*P* = 0.004; risk ratio = 3.779) (33). Charafe-Jauffret and colleagues showed that when the model contains tumor size, SBR grade, and hormonal receptors, MSN was nearly an independent prognostic marker for patients without axillary lymph node involvement (HR = 2.38, 95% CI 0.99–5.56, *P* = 0.052) (39). Donizy et al. (41) found that enhanced MSN immunoreactivity was an independent prognostic factor (*P* = 0.028). In the study of Yu et al., MSN expression has no significant correlation with OS (*P* = 0.452) (45).

**Table 3:** The association of high MSN expression and survival analysis.

Authors	Follow-up (months)	The location of MSN	Outcome	Univariate analyses	Multivariate analyses	Prognostic value
Chotteau-Lelièvre (33)	77.6	–	94 deaths and 126 relapses	OS: *P* = 0.006, RR = 2.95	OS: *P* = 0.004, RR = 3.779	According to the survival analysis, MSN was regarded as an independent adverse prognostic marker for patients with breast cancer.
Charafe-Jauffret (39)	82	Cytoplasm	–	SS: *P* = 0.014, MFS: *P* = 0.014	*P* = 0.052, HR = 2.38, 95% CI 0.99–5.69	MSN not only was a marker of basal breast cancer but also could be a poor prognostic marker for patients.
Charpin (40)	79	–	181 metastases and 32 deaths	*P* = 0.00001^a^ *P* = 0.00002^b^	–	The study reported that MSN had prognostic value in breast cancer.
Donizy (41)	–	–	–	SS: *P* = 0.0079, DFS: *P* = 4.1e-05	–	MSN overexpression would cause shorter cancer-specific survival and disease-free survival.
Wang (42)	ND	–	–	OS: *P* = 0.0263	–	The study demonstrated that MSN was an EMT marker and MSN had prognostic value in patients with breast cancer.
Li (43)	–	–	–	MFS: *P* = 0.0073 RFS: *P* = 0.0313	–	The study reported that high MSN expression was closely related with worse prognosis of patients with BC.
Pei (44)	–	Cytoplasm and membrane	–	5-y OS: *P* = 0.042^c^ OS: *P* = 0.021^c^	–	This research showed that compared with the low MSN expression, high MSN expression would cause reduced overall survival.
Yu (45)	–	Cytoplasm	–	OS: *P* = 0.452, RR = 1.343, 95% CI 0.621–2.904 RFS: *P* = 0.032, RR = 1.762, 95% CI 1.034–2.976	OS: *P* = 0.490, RR = 0.725, 95% CI 0.291–1.806 RFS: *P* = 0.062, RR = 1.7833, 95% CI 0.970–3.276	MSN could be a marker for unfavorable prognosis in patients with ER-positive breast cancer treated with tamoxifen.
Qin (46)	–	–	–	OS: *P* = 0.0017^d^	–	Stronger MSN expression in the TNBC, which elucidated that there was a negative correlation between MSN expression and OS.

P-value <0.05 was considered statistically significant. All survival time was calculated from the date of diagnosis of BCs.

OS, overall survival; RFS, relapse-free survival/recurrence-free survival; DFS, disease-free survival; SS, specific survival; MFS, metastasis-free survival; NS, not significant; RR, relative risk; ND, no data; 5-y, 5-year: HR, hazard ratio; –, not reported.

a P-value indicated the value of MSN in predicting disease outcome in breast carcinomas.

b P-value showed the value of MSN in predicting disease outcome, when ER, PR, and c-erbB-2 were included in breast carcinomas.

c P-value indicated that compared with patients with low MSN expression, patients with strong MSN expression had lower 5-y OS and OS.

d P-value originated from the Nathan Kline Institute (NKI) database contained in an online database (PROGgeneV2), which illustrated that patients with high MSN expression had lower OS than patients with low MSN expression.

### 3.6 Meta-Analysis Results

**Figure 2:**
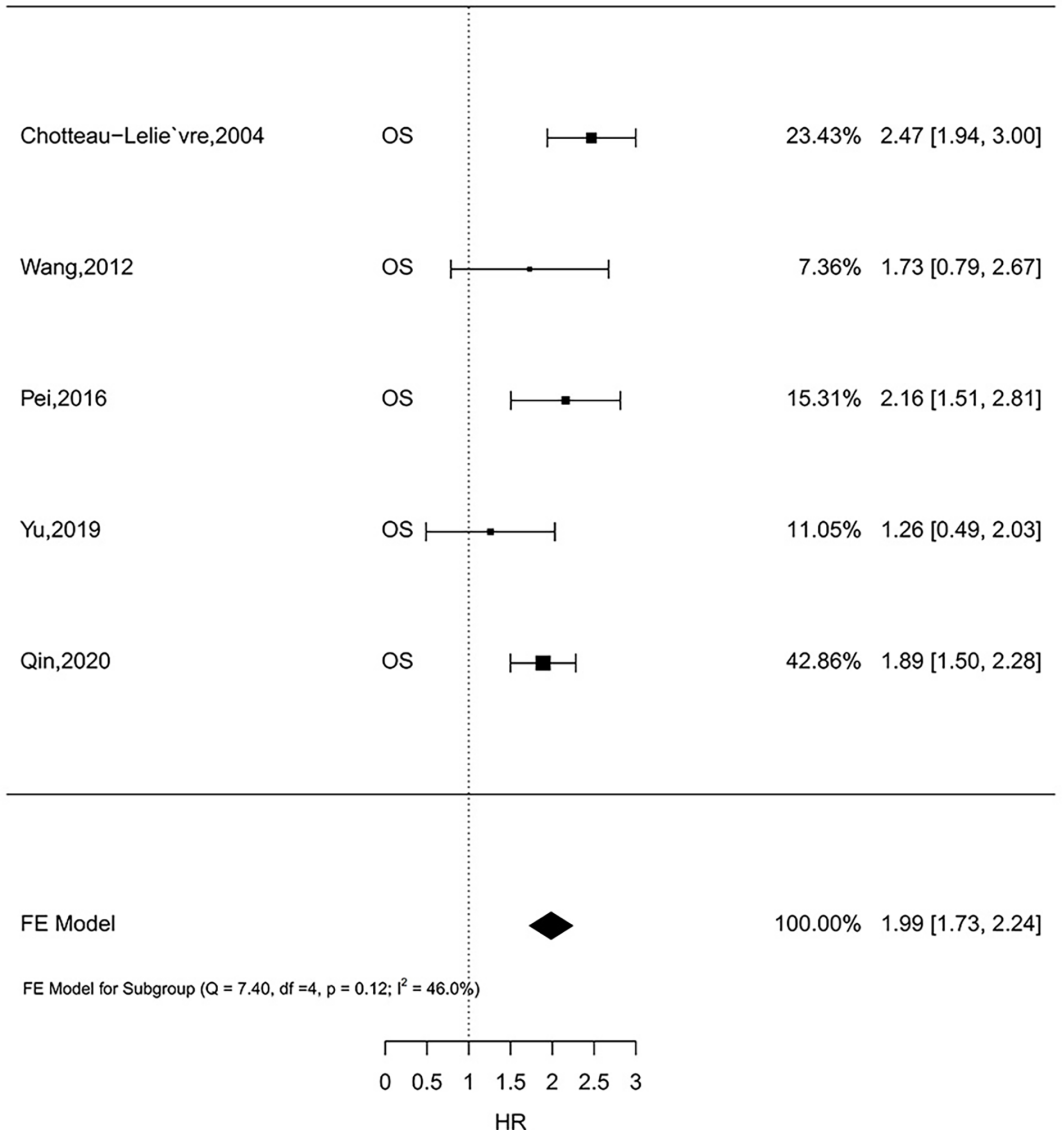
The relationship of MSN expression and endpoints in the GEO datasets, and the results were expressed in terms of hazard ratio (HR) and 95% confidence interval (CI).

Five studies comprising 1,726 patients investigated the prognostic role of MSN expression in breast cancer (33, 42, 44–46). Because there was no heterogeneity (*I* ^2^ = 46.0%, *P* = 0.12), the fixed-effect analysis was applied. Meta-analysis results showed high MSN expression was associated with poor outcomes of breast cancer (HR = 1.99, 95% CI 1.73–2.24) (**Figure 2**). The result in one literature showed that high MSN expression caused poor SS (HR = 1.87, 95% CI 1.45–2.29). Furthermore, a high expression of MSN is strongly associated with a low RFS (HR = 1.86, 95% CI 1.38–2.34). These results suggest that MSN may have a prognostic value in breast cancer patients.

### 3.7 Validation of Meta-Analysis Results

**Figure 3:**
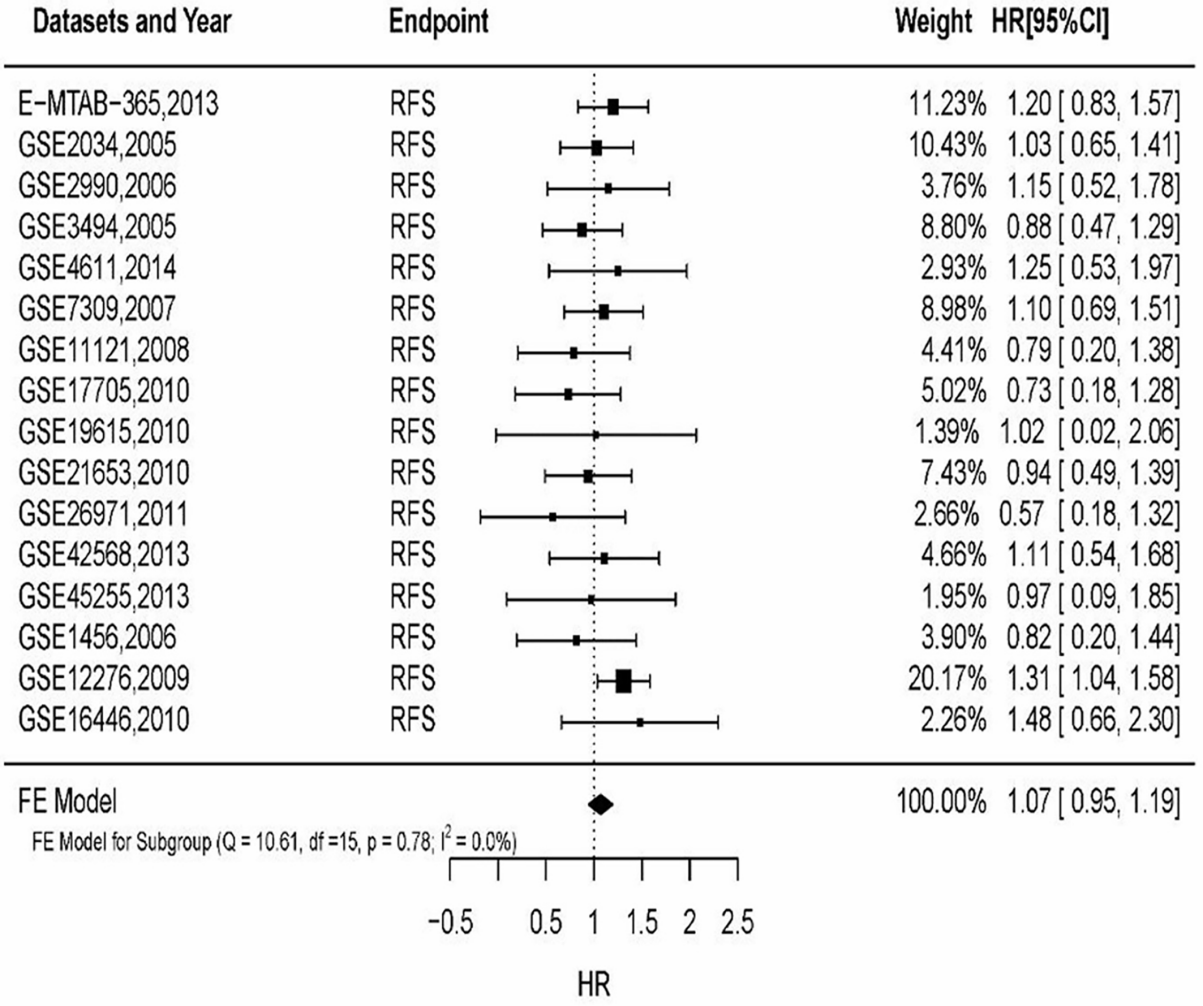
The relationship of MSN expression and endpoints in the GEO datasets, and the results were expressed in terms of hazard ratio (HR) and 95% confidence interval (CI).

By filtering sample size of breast cancer patients, 16 datasets were included to analyze MSN expression in prognosis. The results (**Figure 3**) showed that HR of MSN expression has no heterogeneity (*I* ^2^ = 0%, *P* = 0.78). The results of GEO datasets suggested that high levels of MSN are associated with high risk of death. The datasets validated the literature review.

### 3.8 Publication Bias

**Figure 4:**
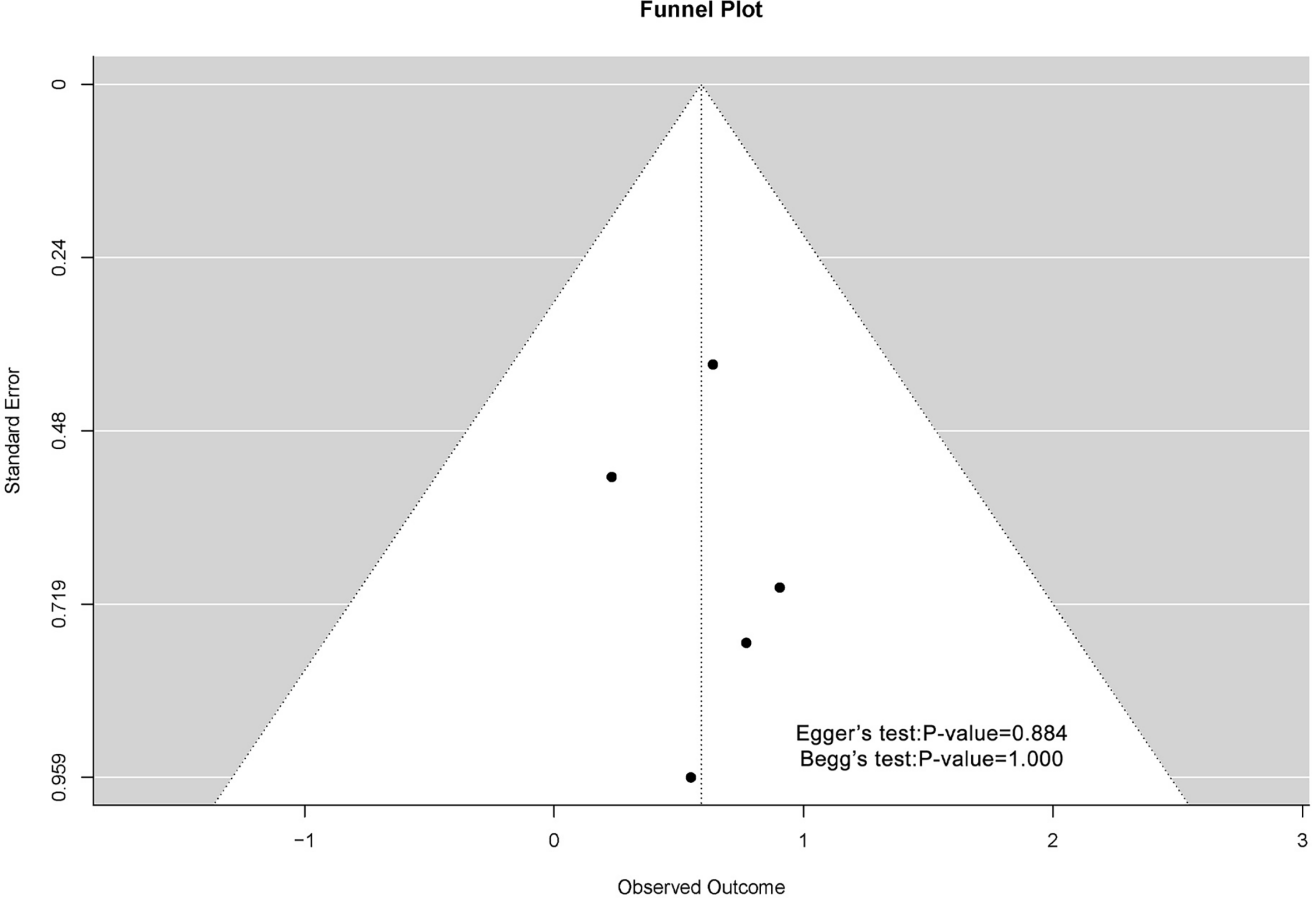
Begg’s funnel plots for the publication bias test of OS.

The funnel plots associated with MSN expression and outcome of breast cancer patients are shown in **Figure 4**. Possibly because of the limitation of literature quantity, the chart was asymmetric on visual examination. The result of Begg’s test showed that *P*-value was greater than 0.05, which meant that there was no publication bias.

### 3.9 Results of the Kruskal–Wallis Test

**Figure 5:**
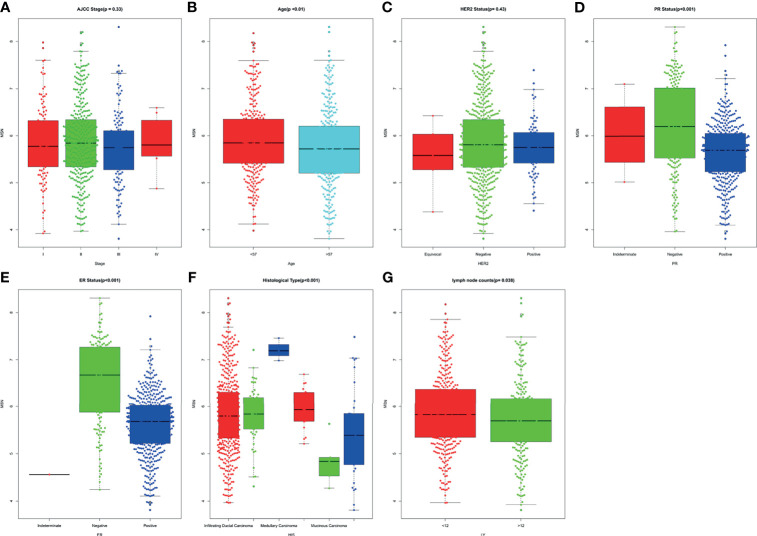
The association of MSN expression and clinicopathological features.

The Kruskal–Wallis test was conducted to evaluate the association of MSN expression with the age at diagnosis, ER status, PR status, HER2 status, histological type, count of lymph node examined, and AJCC stage. MSN expression was not associated with HER2 status and AJCC stage. Compared with patients aged >57 years, the high expression of MSN was significantly associated with patients aged <57 years at diagnosis (*P* < 0.01). Furthermore, patients with ER/PR-negative status had a significantly higher expression of MSN than patients with ER/PR-positive status (*P*_ER_ < 0.001, *P*_PR_ < 0.001). The expression of MSN was significantly correlated with histological type of breast cancer (*P* < 0.001), and we found that when the threshold was 12, MSN expression was closely related with lymph node metastasis (*P* = 0.038) (**Figure 5**).

## 4 Discussion

Here, we noted that high MSN expression correlated with histological grade, ER/PR status, and lymph node metastasis. Our results demonstrated that high MSN expression was negatively correlated with the prognosis of breast cancer, and this was consistent with the result in oral cancer (10, 27), pancreatic cancer (28), and glioma (29, 48). These data indicated that MSN may play an important role in tumorigenesis. Additionally, in the study on ER/PR status, it was shown that ER-positive breast cancer was less aggressive and had better survival than ER-negative breast cancer (49). Compared with ER/PR-positive breast cancer, higher MSN expression was shown in ER/PR-negative breast cancer, which indicated that the ER and PR signaling pathways might be involved in high MSN expression in breast cancer (45, 49). There were prominent relationships between the levels of MSN expression and the therapeutic response of breast cancer. Patients with low MSN expression treated with anthracycline alone or combined with paclitaxel chemotherapy demonstrated a significantly increased RFS than patients with high MSN expression (*P* = 0.027), and patients with low MSN expression treated with tamoxifen obtained better RFS than patients with high MSN expression (*P* = 0.005) (45). Furthermore, it was reported that MSN silencing restored the sensitivity of the p53-mutant cells 1001 to doxorubicin (31). However, there were some studies indicating that the expression of MSN is not associated with the prognosis of breast cancer (32, 50). As for the result that MSN was not related with worse outcome, it may be that the patient cohort was limited with stage II and patients in all stages of breast cancer were not targeted. Besides, the low level of MSN transcripts may not represent the expression of protein levels (45). What is more, there was no specific description of the exact location of the sample on tumor in the original studies, so MSN expression in the center or edge of the lesion may be associated with different results.

MSN expression was associated with metastasis and invasion in various tumors. Our study also found that high MSN expression was negatively correlated with PFS and positively correlated with lymph node metastasis. Related basic research also revealed that MSN promoted the metastasis and invasion of breast cancer. Podoplanin recruits MSN to activate RhoA to promote EMT and facilitate tumor cell invasion and migration (51). Besides, when MSN was silenced in 1001, the 1001 cells reverted from mesenchymal-to-epithelial phenotype and reduced cell migration and invasion (31). These data suggested a close relationship between MSN and EMT. One study showed that talin regulated moesin–NHE-1 recruitment to invadopodia and promoted mammary tumor metastasis (12). Moreover, the loss of MSN expression could promote the invasion and metastasis of breast cancer cells by increasing the transcription level of NM-23 and the secretion of MMP9 and decreasing the expression of metadherin (52). Moreover, one study showed that PR agonists could activate MSN and promote breast cancer cell motility by rapid remodeling of the actin skeleton following MSN activation (53). CD44 is a cell surface adhesion receptor that is widely expressed in most cell types, which belongs to the hyaluronan (HA) receptor family of cell surface glycoproteins (54). One recent study showed that *via* upregulation of p-moesin, CD44 cross-linking increases the malignancy of breast cancer. Moesin knockdown attenuated the promoting effect of CD44 cross-linking on tumor cell invasion and metastasis (55). Recently, Luo et al. (56) proposed a novel mechanism of MSN contributing to tumor invasion and metastasis. ROCK1 increased TMEM16A (a Ca^2+^-activated chloride channel) channel activity through MSN phosphorylation, to promote cell migration and invasion. Studies reported that lymph node metastasis was an important marker for the spread of breast cancer, and it could be a poor marker of prognosis (57, 58). Charafe-Jauffret et al. (39) showed that MSN was related to the rate of metastasis, which suggested that MSN participated in tumor metastasis. Ni et al. (30) also showed that moesin expression was also significantly higher in breast cancer with lymph node metastasis than in breast cancer without lymph node metastasis. Moreover, Yu et al. (45) indicated that the high expression of MSN had significant correlations with positive node metastasis, compared with low expression of MSN (*P* < 0.0001). Together, these results highlight the participation of MSN in the metastasis of breast cancer.

This meta-analysis was performed according to the guidelines of PRISMA (34) and REMARK (35), and the results showed that high MSN expression was strongly associated with poor outcome of breast cancer. According to the Kruskal–Wallis test, the association between MSN expression and histological grade, ER/PR status, HER2 status, lymph node metastasis, AJCC stage, and age at diagnosis was also analyzed. These positive factors contributed to the strengths of this meta-analysis.

The evidence included in the present meta-analysis indicated high MSN expression as a poor prognostic marker in breast cancer. However, there are still some limitations in the present study. First, with the few available studies and the small sample size of patients included in this review, the results might be less powerful. Besides, many articles only described the relationship between MSN and metastasis without data on MSN and survival; therefore, more eligible articles could not be included for quantitative analysis. In addition, because some HRs were calculated indirectly by the data extracted from the literature, these data were less reliable than direct data from the original literature.

## 5 Conclusions

By analyzing the literature and meta-analysis results, we found that high MSN expression correlated with more aggressive clinicopathological features and poorer prognosis in patients compared with lower MSN expression. In addition, we need to expand the patient cohort with additional studies to confirm our results.

## Data Availability Statement

Publicly available datasets were analyzed in this study. These data can be found here: GEO database and TCGA-BRCA database (https://portal.gdc.cancer.gov/).

## Author Contributions

YL and XH determined the study direction. CL, YL, and ZB provided the research methods. XH, QY, and CL performed the literature retrieval and data management. ZB, QY, YL, and XH analyzed and interpreted the results. XH wrote the manuscript. All authors contributed to the article and approved the submitted version.

## Funding

This study was supported by a grant from the National Natural Science Foundation of China (No. 11575262).

## Conflict of Interest

The authors declare that the research was conducted in the absence of any commercial or financial relationships that could be construed as a potential conflict of interest.

## Publisher’s Note

All claims expressed in this article are solely those of the authors and do not necessarily represent those of their affiliated organizations, or those of the publisher, the editors and the reviewers. Any product that may be evaluated in this article, or claim that may be made by its manufacturer, is not guaranteed or endorsed by the publisher.
