# Supplementary material for: High Moesin Expression Is a Predictor of Poor Prognosis of Breast Cancer: Evidence From a Systematic Review With Meta-Analysis
Source: Front Oncol. 2021 Nov 26;11:650488. doi: 10.3389/fonc.2021.650488 (PMC8660674; doi:10.3389/fonc.2021.650488)
Supplement: Supplementary file 1 [file Table_1.docx]

Supplementary Material

**Table S1** Search Strategy

Database: PubMed

| Terms |  | Keywords |
| --- | --- | --- |
| Moesin | #1 | membrane-organizing extension spike protein |
|  | #2 | Msn protein |
|  | #3 | moesin protein |
|  | #4 | MSN protein |
| Breast cancer* | #5 | Breast Neoplasm |
|  | #6 | Breast Tumor* |
|  | #7 | Breast Cancer* |
|  | #8 | Mammary Cancer* |
|  | #9 | Malignant Neoplasm of Breast |
|  | #10 | Breast Malignant Neoplasm* |
|  | #11 | Malignant Tumor of Breast |
|  | #12 | Breast Malignant Tumor* |
|  | #13 | Cancer of Breast |
|  | #14 | Cancer of the Breast |
|  | #15 | Mammary Carcinoma* |
|  | #16 | Human Mammary Carcinoma* |
|  | #17 | Human Mammary Neoplasm* |
|  | #18 | Breast Carcinoma* |
|  | #19 | #1 OR #2 OR #3 OR #4 |
|  | #20 | #5 OR #6 OR #7 OR #8 OR #9 OR #10 OR #11 OR #12 OR #13 OR #14 OR #15 OR #16 OR #17 OR #18 |
|  | #21 | # 19 AND #20 |

**TABLE S2** Evaluation criteria used to assess the quality of records.

| Checklist Items | Criteria* |
| --- | --- |
| 1. Patient samples | Cohort (retrospective or prospective) study with a well-defined study population with information such as the number of the studied patients, source of sample, study period, follow-up time. |
| 2. Clinical data of the cohort | The clinical data including age, clinical stage of cancer, histological grade, ER/ PR status and Lymph node involvement, was provided. |
| 3. Assay methods | Well-described staining protocol or referred to original paper with information such as primary antibody name, dilution, company. The Cutoff value of the area stained after which it is to be considered positive, was well described. |
| 4. Prognostics | The endpoints of the survival analyses were defined (e. g. overall survival, relapse free survival, disease-free survival and metastasis-free survival). |
| 5. Statistical analysis | Estimated effects (HR, CI) were describing the relationship between the MSN expression and the BC outcome was provided. Adequate statistical analysis was performed. |
| 6. Classical prognostic factors | The classical prognostic factors were reported in original articles. |

Abbreviations: HR, hazard ratio; CI, confidence intervals.

* The criteria was adapted from the reporting recommendations for tumor marker prognostic studies (REMARK) guidelines (35).

| No | References | Reasons for exclusion |
| --- | --- | --- |
| 1 | Carmeci C, Thompson DA, Kuang WW, Lightdale N, Furthmayr H, Weigel R J. Moesin expression is associated with the estrogen receptor-negative breast cancer phenotype. Surgery (1998) 124, 124(2): 211-217. | Without survial  analysis |
| 2 | Ni X, Shao Z. Relationship between moesin expression and breast cancer metastasis. Fudan University Journal of Medical Sciences ( 2013) 40 (6): 679-684. | Without survial  analysis |
| 3 | Beaty BT, Wang Y, Bravo-Cordero JJ, Sharma VP, Miskolci V, Hodgson L, Condeelis J, et al. Talin regulates moesin-NHE-1 recruitment to invadopodia and promotes mammary tumor metastasis. Journal of Cell Biology (2014) 205 (5): 737-751. | Without survial  analysis |
| 4 | Abdel-Rahman WM, Alam F, Mezhal F, Ayad MS, El-Serafi A, El Hassasna H. Study of Moesin regulation and effects in breast cancer cells. European Journal of Cancer (2016) 61:S59-S59. | Without survial analysis |
| 5 | Hong H, Yu H, Yuan J, Guo C, Cao H, Li W, et al. MicroRNA-200b Impacts Breast Cancer Cell Migration and Invasion by Regulating Ezrin-Radixin-Moesin. Medical Science Monitor (2016) 22:1946-1952. | Without survial  analysis |
| 6 | Alam F, Mezhal F, El Hasasna H, Nair VA, Aravind SR, Saber AM, et al. The role of p53-microRNA 200-Moesin axis in invasion and drug resistance of breast cancer cells. Tumour biology : the journal of the International Society for Oncodevelopmental Biology and Medicine (2017) 39 (9):1010428317714634-1010428317714634. | Without survial  analysis |
| 7 | Bartova M, Hlavaty J, Tan Y, Singer C, Pohlodek K, Luha J, et al. Expression of ezrin and moesin in primary breast carcinoma and matched lymph node metastases. Clinical & Experimental Metastasis, 2017, 34 (5): 333-344. | Without survial  analysis |
| 8 | Wu Q, Chen D, Luo Q, Yang Q, Zhao C, Zhang D, et al. Extracellular matrix protein 1 recruits moesin to facilitate invadopodia formation and breast cancer metastasis. Cancer Lett (2018) 437: 44-55. | Without survial  analysis |

**TABLE S3** List of the excluded studies and the reasons for exclusion.

**TABLE S4** List of the included studies.

| No. | References |
| --- | --- |
| 1 | Chotteau-Lelie`vre A, Re´villion F, Lhotellier V, Hornez L, Desbiens X, Cabaret V, et al. Prognostic Value of ERM Gene Expression in Human PrimaryBreast Cancers. Clinical Cancer Research (2004) 10 (1):7297–7303. |
| 2 | Charafe-Jauffret E, Monville F, Bertucci F, Esterni B, Ginestier C, Finetti P, et al. Moesin expression is a marker of basal breast carcinomas. Int J Cancer (2007) 121:1779-1785. doi:10.1002/ijc.22923. |
| 3 | Charpin C, Giusino S, Secq V, Carpentier S, Andrac L, Lavaut M, et al. Quantitative immunocytochemical profile to predict early outcome of disease in triple-negative breast carcinomas. International Journal of Oncology (2009) 34 (4): 983-993. doi:10.3892/ijo_00000224. |
| 4 | Donizy P, Halon A, Matkowski R. Elevated moesin immunoreactivity is a new unfavorable prognostic factor in breast cancer patients treated with CMF based chemotherapy. Annals of Oncology ( 2011) 22: ii62-ii63. |
| 5 | Wang CC, Liau JY, Lu YS, Chen J, Yao Y, Lien H. Differential expression of moesin in breast cancers and its implication in epithelial-mesenchymal transition. Histopathology (2012) 61(1): 78-87.doi: 10.1111/j.1365-2559.2012.04204.x |
| 6 | Li X, Roslan S, Johnstone CN, Wright JA, Bracken CP, Anderson M, Bert AG, et al. MiR-200 can repress breast cancer metastasis through ZEB1-independent but moesin-dependent pathways. Oncogene (2014) 33 (31): 4077-4088. doi:10.1038/onc.2013.370. |
| 7 | Pei XJ, Xue XF, Zhu YL, et al. Expression and clinical significance of moesin and E-cadherin in invasive carcinoma of breast, no specific type. Zhonghua Bing Li Xue Za Zhi (2016) 45 (8):550-555. |
| 8 | Yu L, Zhao L, Wu H, Zhao H, Yu Z, He M, Jin F, We M, et al. Moesin is an independent prognostic marker for ER-positive breast cancer. Oncol Lett (2019)17:1921-1933. doi: 10.3892/ol.2018.9799. |
| 9 | Qin Y, Chen W, Jiang G, Zhou L, Yang X, Li H, et al. Interfering MSN-NONO complex-activated CREB signaling serves as a therapeutic strategy for triple-negative breast cancer. Science advances (2020) 6 (8): eaaw9960. |
